# Vectorial strain gauge method using single flexible orthogonal polydimethylsiloxane gratings

**DOI:** 10.1038/srep23606

**Published:** 2016-03-23

**Authors:** Hao Guo, Jun Tang, Kun Qian, Dimitris Tsoukalas, Miaomiao Zhao, Jiangtao Yang, Binzhen Zhang, Xiujian Chou, Jun Liu, Chenyang Xue, Wendong Zhang

**Affiliations:** 1 Science and Technology on Electronic Test & Measurement Laboratory, North University of China, Taiyuan, Shanxi, 030051, China; 2 Department of Applied Physics, National Technical University of Athens, GR-15780 Zografou, Greece

## Abstract

A vectorial strain gauge method using a single sensing element is reported based on the double-sided polydimethylsiloxane (PDMS) Fraunhofer diffraction gratings structures. Using O_2_ plasma treatment steps, orthogonal wrinkled gratings were fabricated on both sides of a pre-strained PDMS film. Diffracted laser spots from this structure have been used to experimentally demonstrate, that any applied strain can be quantitatively characterized in both the *x* and *y* directions with an error of less than 0.6% and with a gauge factor of approximately 10. This simple and low cost technology which is completely different from the traditional vectorial strain gauge method, can be applied to surface vectorial strain measurement and multi-axis integrated mechanical sensors.

Vector mechanical sensors, which can sense mechanical parameters providing information about both their strength and their direction, have been widely applied for acoustic and force sensing in large structures such as submarines, spacecrafts or bridges and buildings but they are also investigated for the development of artificial skin^1^,^2^,^3^. To reduce the size and cost of these sensors and to provide improved chip integration, researchers have developed different sensing methods using new materials, techniques and algorithms over the last decade^4^,^5^,^6^.

To achieve the vectorial strain gauge, various assembly technologies have been developed. Yang^7^ reported a self-powered wind vector sensor system for wind speed and direction detection using four same triboelectric nanogenerators arranged along four different directions to sense the magnitude and direction of wind signals. Zhao^8^ fabricated a high sensitivity vectorial strain gauge by assembling two same flexible microelectrodes in cross direction, which can be tuned to detect the magnitude and orientation and were appropriate for wearable and conformal electronics. However, the above approach was prone to errors which were related to the assembly of two or more of sensitive elements and the complexity of the fabrication process being at the same time of relatively large size.

To reduce the cross direction and assembly error as well as the chip size, researchers are investigating methods for single-chip integration. Another proposed solution for single-chip vectorial mechanical parameter sensing was the use of a novel structural design. Jing *et al.*^4^ reported a robust and self-powered kinematic vector sensor by decoupling information from the two same elements, which were placed in cross to obtain the vectorial signals. Kang^9^ has used twenty-four strain gauges in one device to optimize the design of a mechanically decoupled six-axis force/torque (F/T) sensor through the minimization of cross coupling error. However, this device required complicated micro/nano manufacturing and packaging processes to be realized.

Because of their low cost, high manufacturing precision and uniformity, an increasing number of optical components were investigated and developed using grating structures based on flexible substrate materials (e.g. polydimethylsiloxane (PDMS) and polymethylmethacrylate (PMMA)), including polarisers and optical filters, among others. Shishido^10^ proposed a facile method for two-dimensional evaluation of surface strains in largely bending films based on surface-labelled gratings used for spatial strain mapping of samples with complex curvature which can be applied to non-transparent objects. Xie^11^ presented a six-spot grating diffraction strain gauge to measure mechanical behaviour (displacement and rotation) at high-temperature for deformations in two directions. These structures have also been studied for application to sensing of quantities such as strain/stress, acceleration and acoustic waves^12^,^13^,^14^.

Yang *et al.*^15^ have demonstrated a G-Fresnel device based on double side grating formation on PDMS, which has dual functionality operating as diffraction grating and as Fresnel lens and can be integrated as an optofluidic device for on-chip spectroscopy.

In the literature, vectorial sensing was demonstrated by using two types of approaches: one that was based on multiple elements assembled together to form the sensing element^7^,^8^ and others that use structure decoupling^9^. In this paper, we demonstrate a strain/stress vector sensing method based on a single element, the orthogonal optical grating structure, which was formed on both sides of a PDMS substrate. This geometric configuration results in an opposite movement of the diffractive spot created by a laser beam crossing the structure as a function of stress. The relative displacement and intensity of these spots can be then used to estimate the vectorial strain/stress information. Using this structure, the applied strain can be quantitatively characterized in both the *x* and *y* directions, with an error of 0.6%, and with a gauge factor of approximately 10. This work represents an important step towards practical application of single-chip integrated vector sensor systems.

Compared with previous attempts to use polymer planar waveguide grating structures assembled from two same elements^16^ or from two same detectors^17^, our results demonstrate much smaller error compared to the assembling errors. It is also simpler both in terms of system design but also in tterms of fabrication processing, allowing for a detailed study of the performance of the integrated component and the demonstration of vectorial strain application. We attribute this to single-chip integration with non-coupling of the two gratings made with controlled periodicity. To diminish the error from the measurement setup, MATLAB software has been used to real-time track and position the nine diffraction spots and to align the central spot of the images at every time.

## Results

### Orthogonal PDMS grating and strain gauge characterization

**Figure 1 f1:**
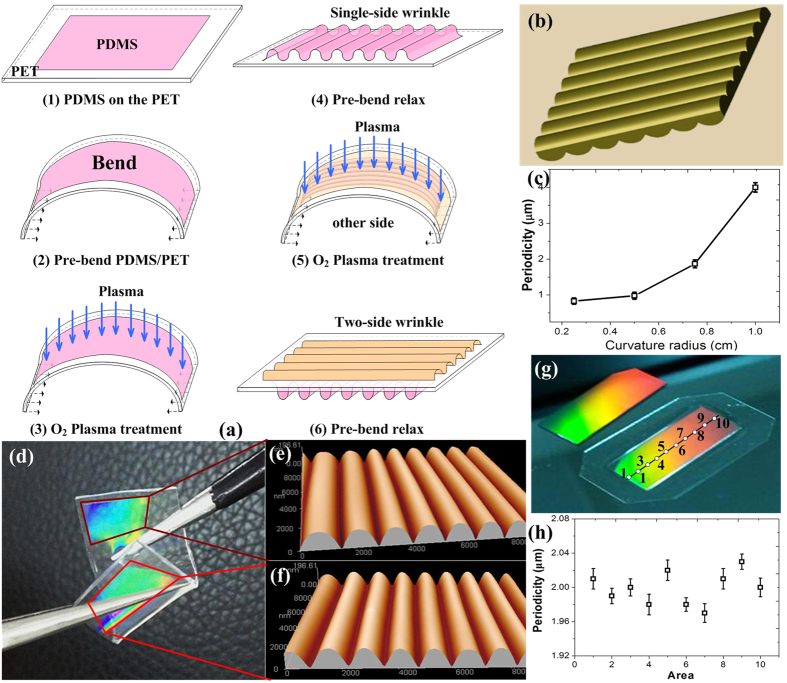
PDMS grating fabrication and characterizations. (**a**) Schematic diagram of the fabrication process of double-side orthogonal grating structure on the surface of PDMS. In step 5 PDMS was turned by 90°. (**b**) The model of double-side orthogonal grating structure. (**c**) The periodicity vs the curvature radius. (**d**) Double-side orthogonal grating. (**e,f**) AFM image of grating structure for both sides. (**g**) The single side grating. (**h**) The uniformity of periodicity for the samples.

The fabrication process is presented in Fig. 1. Oxygen plasma treatment was used to modify the hydrophobic PDMS with hydrophilic functionalities. A SiO_x_ layer and hydrophilic groups (e.g. -OH) have been thus formed on pre-bent PDMS substrates by the O_2_ plasma. When the strain of PDMS exceeds a critical value, the PDMS substrate self-assembles to form folded grating structures after bent relaxation^18^. By controlling the side on which the plasma treatment was applied to the PDMS surface, double-sided grating structures were fabricated with periodicity of approximately 2 μm. As shown in Fig. 1g[Fig f1]h, the periodicity of the grating was uniform with of (2 ± 0.1) μm period on the whole surface of the sample. Desired periodicity of the gratings was achieved by tuning the applied pre-bent and plasma conditions and can be calculated as introduced in our previous studies^18^,^19^.

**Figure 2 f2:**
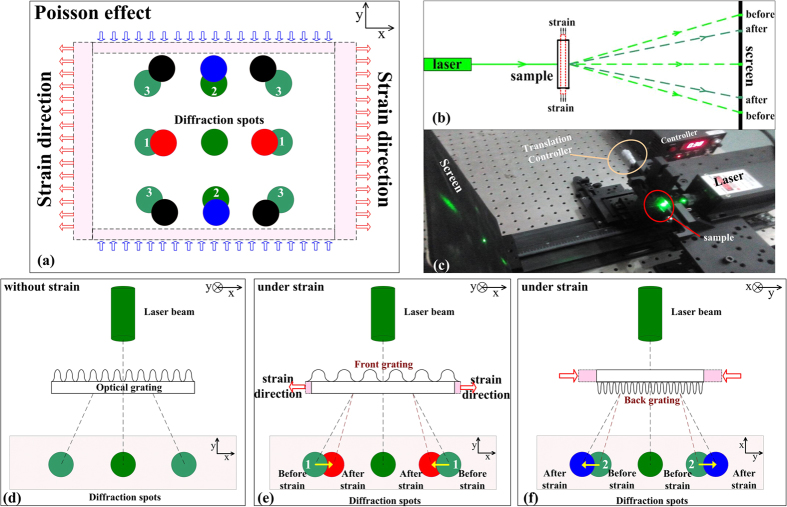
The strain induced diffraction spot movement characterizations. (**a**) The spot moving as a function of strain (**b**) The optical path vs applied strain (**c**) The optical measurement setup (**d**) Optical diffraction without strain (**e**) Optical diffraction with strain vertical to grating (**f**) Optical diffraction with strain along the grating direction.

Strain gauge characterization tests were first performed, as shown in Fig. 2. The double-sided PDMS grating was fixed to a translation stage (as shown in Fig. 2c). The strain was applied by controlling the micrometer located on the controller. The strain was initially applied either along or across the PDMS grating.

According to the theory of multi-slit Fraunhofer diffraction, the grating periodicity changes as a function of stretching, thus resulting in the first-order movement of the diffraction spot shown in Fig. 2a. Without the applied strain, the diffraction spots were distributed evenly over nine positions, as indicated by the green spot in Fig. 2a[Fig f2]d.

The PDMS gratings on the two sides of the substrate were orthogonal. When the applied strain is along the direction of the grating in one side, this strain is perpendicular to the direction of the grating on the other side of the substrate. In our work, the direction of grating at the front side was defined vertical to *x* axis and the back side grating was then parallel to the *x* axis. Under the applied strain along the *x* axis, two phenomena occur, as shown in Fig. 2e[Fig f2]f. As the applied strain was perpendicular to the front grating direction, the length of PDMS increased and the front grating periodicity was consequently increased, resulting in the movement of the diffraction spots ‘1’ closer to the centre, as indicated by the red spots in Fig. 2e. At the same time, due to the Poisson effect, the width of PDMS has shrunk, resulting to a reduced periodicity of the back side grating. As indicated by the blue spots in Fig. 2f, the diffracted laser spots ‘2’ from the back grating have moved away from the centre. The spots ‘3’ have a complex movement that as we demonstrate later in this paper was the resultant of movements of the spots ‘1’ and ‘2’ and indicated by the black spots in Fig. 2a.

**Figure 3:**
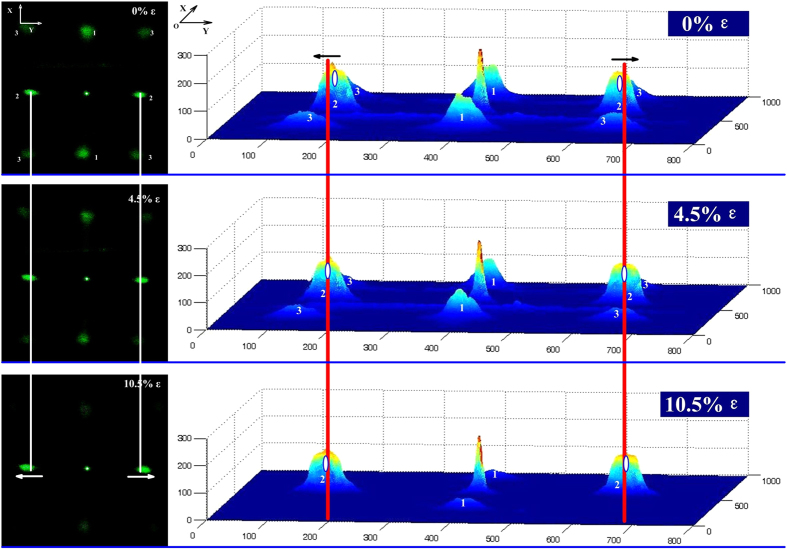
Calculation of strain from the diffracted laser spot using MATLAB.

**Figure 4:**
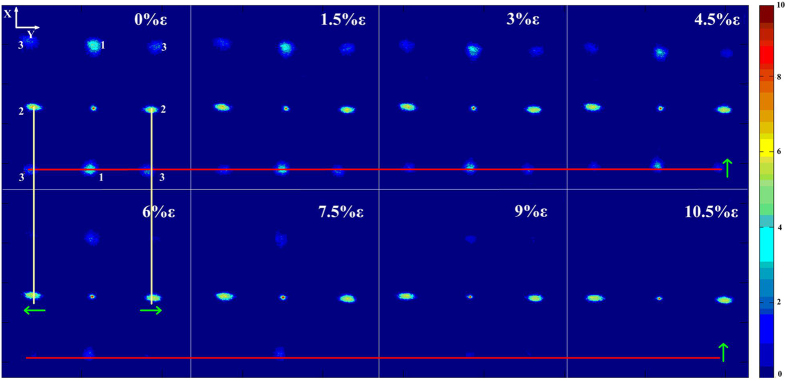
Photographs of laser diffractions through the double-side orthogonal PDMS grating with different applied strain.

To quantitatively characterize the strain gauge performance of the fabricated double-sided PDMS gratings, the home-made characterization system shown in Fig. 2c was used to analyse the diffraction laser spot images, as shown in Figs 3 and 4. We underline that in this work, a tensile strain was applied along the *x* axis, thus being perpendicular to the direction of the grating on the front side of the sample; and at the same time parallel to the direction of the rear side grating.

As shown in Fig 2b, the light beam going from the front side grating through the sample diffracts the three beams to form the nine spots on the screen. When strain was applied, all of the diffracted light beams were moving except of the central beam which has been used to locate the diffraction spots under the applied strain in this method. Stretching of the sample was assumed the same in any directions, such that the front and back surface were deformed in the same manner.

The images were processed using MATLAB which allowed the estimation of both the movement and the intensity variations of the laser spots. Figure 3 shows the process for calculation of the strain from the diffracted laser spots. The intensity distributions of the diffracted laser spots were calculated under different applied strains. By comparing the positions of the peaks of these laser spots, the movements of spots ‘1’, ‘2’ and ‘3’ can all be calculated. Also, by integration of the region containing the spots, the variations in the spots intensity were also characterized. The combination of the movement and intensity variations of the diffracted laser spots provided a characterization of the strain gauge performance of the double-sided gratings.

Figure 4 shows the movement of the diffracted laser spots. Under different applied strains, it was observed that, the spot at the centre always maintained its position, but the eight surrounding spots moved as described below. As shown in Fig. 4, the spot denoted by ‘1’ was formed due to diffraction from the grating perpendicular to the strain on the front side and it has moved closer to the central spot because of the increased grating periodicity on this side. In contrast, the spot denoted by ‘2’ was formed by diffraction from the grating parallel to the strain on the rear side, and it has moved away from the centre because of the reduced grating periodicity on this side. The movement of spots denoted by ‘3’ embrace the movement information of both spots ‘1’ and ‘2’. In addition, the intensities of all laser spots decreased with increasing strain.

Using this method, under the applied strain, the front grating of the sample has an increase in period and a decrease in height. In contrast, the periodicity and height of the back grating changed with the opposite trend. This can be quantitatively calculated as follows^20^,^21^,^22^:


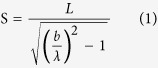


where 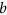 was the initial value of grating periodicity measured at 2 μm, the laser wavelength 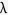 was 532 nm, 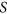 was the distance between the zero-order and first-order diffraction spot, and the distance 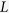 between the sample and the screen was 10 cm.

However, due to the Poisson effect, the thickness of sample is reduced under applied tensile strain as shown in the Fig. 2b. In this paper, the PDMS thickness change was about 0.05 mm under length change of 10.5%, as calculated by the Poisson ratio of 0.48 for the PDMS. So, compared to the distance of 10 cm between the sample and the screen, the front grating was moving closer to the screen and the back grating was moving away from the screen by a very small relative distance change of 0.025% under the maximum strain, which can barely affect this method to sense vectorial strain.

The height *h* of the grating versus the grating periodicity being given by:


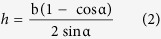



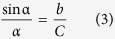


where, 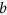 was the modified grating periodicity, C the length of sample being 20 mm, 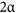 was the curved radian of the grating.

Meanwhile, the intensity can be calculated by^20^,^21^,^22^:


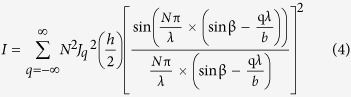



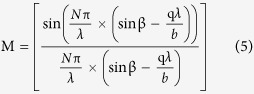


where, 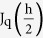 was the first kind Bessel function^23^, 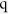 was the diffraction grades, 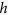 was the height of grating, *N* was the number of gratings, 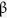 was the diffraction angle, 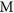 was the diffraction factor, which was used to point out the location of the diffraction spot.

### Strain induced grating structural variation

**Figure 5 f5:**
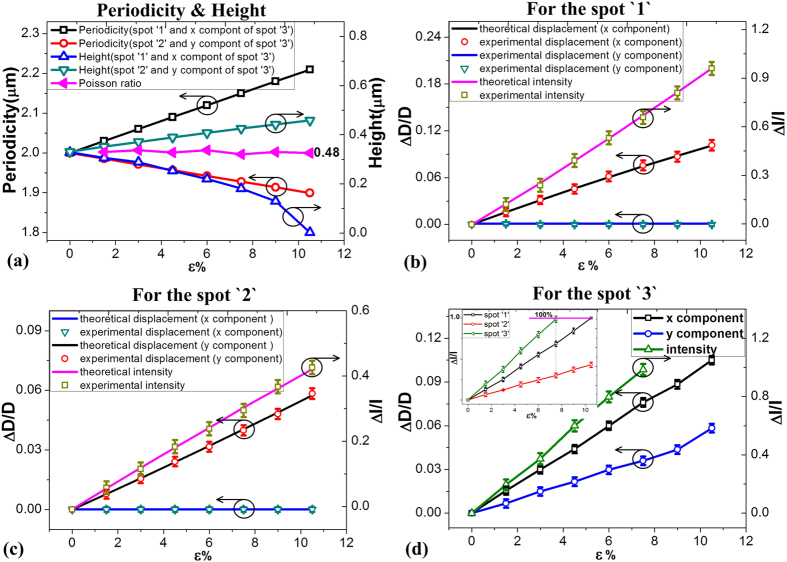
The strain induced optical diffraction characterizations. (**a**) Strain induced grating periodicity and height variations on the two sides. (**b**) Strain induced displacement and intensity variations of spot ‘1’. (**c**) Strain induced displacement and intensity variations of spot ‘2’. (**d**) Strain induced displacement and intensity variations of spot ‘3’.

The grating periodicity and height were calculated using Eqs (1, 2, 3), as shown in Fig. 5a. The front grating (for the spot ‘1’), because it was oriented to be perpendicular to the applied strain, showed increasing periodicity from 2 μm to 2.21 μm when the strain increased to 10.5%, but decreased height from 0.4 μm to 0 μm. For the back grating (for the spot ‘2’), because it was oriented in the same direction as the applied strain, the opposite phenomena were observed. The periodicity of the back grating decreased from 2 μm to 1.89 μm, while its height increased from 0.4 μm to 0.48 μm.

Because of the Poisson effect, the relative changes in the sample were larger along the direction of the applied strain than those in the perpendicular direction. For the front grating, since the tensile direction was along the direction of the applied strain, the relative change in periodicity was 0.21 μm, which was larger than the change of 0.11 μm under the vertical applied strain for the back grating, with the ratio of these two being equal to 0.48, following the Poisson ratio of PMDS. Also, the relative change in height was 0.4 μm for the front grating, which was greater than the change of 0.08 μm for the back grating. These phenomena can be used to support the relative changes in the displacement and intensity of each of the diffraction spots.

### Strain-induced diffraction spot movement and intensity characterizations

To compare the results of the calculated strain from the measured data with the applied strain values, we have combined the relative laser spot movement distance and the intensity change by measuring ten samples, ten times each. As shown in Fig. 5b, under strain of 10.5%, the *x* component of relative change ratio of displacement for the spot ‘1’ has increased to 0.101, while, the *y* component of relative change ratio was zero. Meanwhile, for the spot ‘2’, the *x* component of relative change ratio of displacement was zero, but it has increased to 0.058 along the *y* axis under strain of 10.5% (as shown in Fig. 5c). Compared to spot ‘2’, the spot ‘1’ has shown higher relative change ratio of displacement which was attributed to the greater changes in periodicity that occur for the front grating when compared with those for the back grating under strain applied to the perpendicular direction (*y*-axis). Similarly, the results can also be verified by an intensity test.

As shown in Fig. 5b[Fig f5]c, clear reduction of the intensities of spot ‘2’ was observed. The relative intensity variable for the spot ‘1’ increased to 0.91 (shown in Fig. 5b), which was greater than the corresponding value of 0.43 for the spot ‘2’ (shown in Fig. 5c), due to the greater changes in grating height for the spot ‘1’ than that for spot ‘2’. The height of grating for spot ‘1’ have been stretched to flat surface condition, while the height have been increased for spot ‘2’. However, when compared with the displacement variables, the gauge factor (which was calculated by the equation 
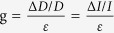
) increased by two times, which indicates that the gauge factor would be more sensitive for strain/stress sensing in practical applications.

### Strain calculation method

As shown in Fig. 2a the displacement of each of the spots ‘3’ has both an *x* component (due to the spot ‘1’, from the front grating) and a *y* component (due to the spot ‘2’, from the back grating). As shown in the inset of Fig. 5d, the *x* component increased to 0.101 which has the same value as the *x* component of the spot ‘1’, and the *y* component increased to 0.058, which has the same value as the *y* component of spot ‘2’. Therefore, the relative change in periodicity in the front and back gratings can be reverse-calculated using Eqs 1, 2, 3.

The ratio of periodicity variation between the two sides was 0.48, which was the same as the Poisson ratio of PDMS (as shown in Fig. 5a). The *x* components of spot ‘3’ were the same with those of the spot ‘1’ and the *y* components were the same with those of the spot ‘2’ i.e the spot ‘3’ movement was the resultant of *x* and *y* movements of spots ‘1’ and ‘2’ respectively. Also, the relative intensity variable of spot ‘3’ was the sum of that of spot ‘1’ and ‘2’, as shown in the inset of Fig. 5d. It then becomes clear that there was a one to one correspondence between all spot positions and intensities with strain direction at least when this was applied along the *x* or *y* axis.

We therefore conclude that the variations in both the intensity and movement of the three types of spot (“1”, “2”, and “3”) can be used to develop a methodology for the calculation of the strain gauge when the applied strain has an unknown direction. In such a methodology the complex diffraction spots pattern can be used to determine the vectorial direction information, and the displacement and intensity variations can be used to estimate the magnitude of the applied signals using the spot ‘3’. These phenomena offer a potential method for vectorial signal sensing using a single element.

### Vectorial strain gauge performance characterization

Using the methods described above, the vectorial strain gauge performance was then characterized using the three types of spots. All samples, equipment and the control system described above were used to study methods of vectorial strain/stress sensing.

**Figure 6:**
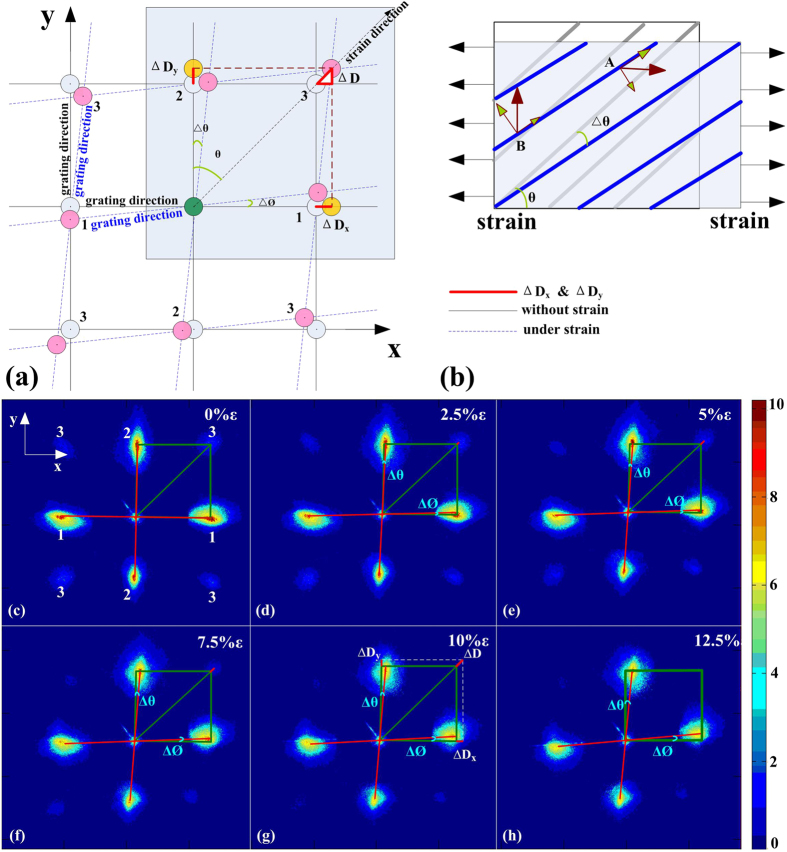
Vectorial strain characterizations. (**a**) The spot moving versus the strain/stress with direction of 45°. (**b**) The corresponding strain/stress model. (**c–h**) the vertical grating with an angle variable of 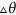 and the horizontal grating with an angle variable of 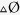.

Figure 6 shows a schematic diagram in the case where the strain/stress was applied in an arbitrary direction. Because of the direction of the applied strain/stress, the grating structures on both sides have been tilted. Spots ‘1’ and ‘2’ have moved out from the *x* or *y* axis and this has resulted in further movement of the spot ‘3’, which can be more sensitive for strain/stress sensing. The displacement of the spots ‘3’ were denoted by 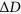, and can be analysed to the *x* and *y* components denoted by 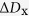 and 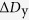 respectively which depend on the direction and the magnitude of the applied strain. The variations in the spot ‘3’ embrace vectorial information about the strain/stress.

The corresponding strain/stress model was shown in Fig. 6b. The sample becomes longer or narrower under applied strain/stress according to Poisson’s theory^24^. The grating dimensional change was a function of the composite strain for both stretching and compression. The strain/stress vector that was projected in both the *x* direction and the *y* direction can be calculated using the algorithm described below.

The magnitude of strain/stress can be calculated as:


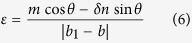



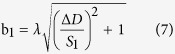



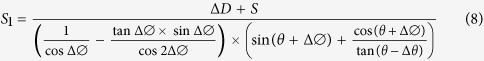



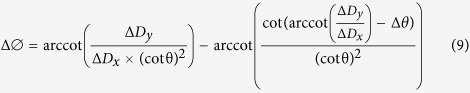


The direction of strain/stress can be calculated as:


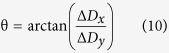


where, 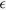 was the strain, 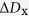 and 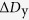 were the displacement components along the *x* and *y*, respectively, 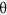 was the angle between the strain direction and grating direction (shown in Fig. 6a), the m and n were the length and width of the sample respectively. Poisson’s ratio 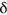 was 0.48, 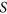 was the distance between the zero-order and the first-order diffraction spot. 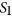 was the relative change of 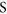 under the strain, 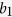 was the relative changes of b, 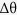 was the angle variable for the vertical grating and 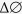 was the angle variable for the horizontal grating under the strain as shown in Fig. 6.

To prove the feasibility of our method we have used an applied strain at 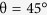 to the grating; we then gradually applied stress along this direction and six group data were tested. As shown in Fig. 6, the eight spots change position as a function of the vectorial strain/stress. The spots ‘3’ move along the direction of the strain/stress. The relative displacements of the spots ‘3’ have been marked as ‘
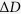
’ in red font, and the *x* and *y* components have been calculated to be ‘
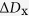
’ and ‘
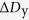
’ respectively, as shown in Fig. 6.

**Figure 7:**
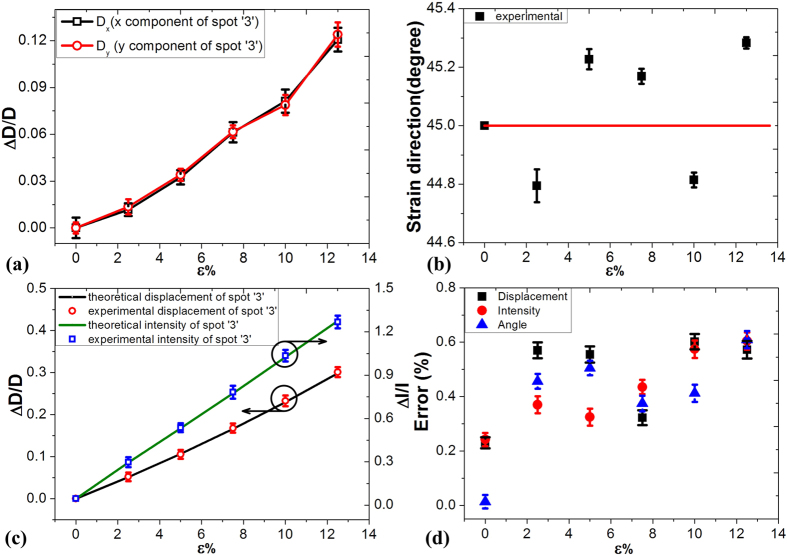
Calculation of the strain induced optical diffraction. (**a**) The *x* component and *y* components of displacement for the spot ‘3’ under applied strain. (**b**) The angle under applied strain. (**c**) Displacement and intensity under applied strain (**d**) Calculated error.

The displacement and intensity of ‘3’ diffraction spot can be used to calculate the magnitude and direction of the applied strain/stress. The 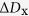 and 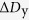 components of the displacement have been calculated using MATLAB software, as shown in Fig. 7a. The direction of the strain was calculated to be approximately 45° using Eq. 10, as shown in Fig. 7b. Also, the magnitude of the applied strain on the sample can be calculated using Eqs 6, 7, 8, 9. Under strain of 12.5%, the relative change ratios of the displacement and the intensity have increased to 0.28 and 1.32, respectively (as shown in Fig. 7c, respectively). The gauge factor calculated from the displacement and intensity data of ‘spot 3’ is 2 and 10 respectively.

As shown in Fig. 7d, the maximum relative error between the calculated strain and the applied strain was 0.6%. To obtain this value we have measured 10 samples, 10 times each. From Fig.7d we observe that the calculated mean value deduced from the 10 samples does not differ from the applied strain. However there is a deviation from this mean strain value over the 10 samples, observed for all of the three variables used, that has a maximum equal to 0.6% which is considered as the error (inaccuracy) of the proposed method. The measurement results show quite good agreement with the theoretical results. Therefore, this method has been successfully used to sense vectorial strain/stress signals using a single element.

## Discussion

A low-cost, simple and manufacturable technology was demonstrated for fabrication of an orthogonal optical grating structure with periodicity of 2 μm and height of 0.4 μm on both sides of a PDMS substrate. This structure can be used to develop a promising single element sensor for vectorial strain/stress monitoring based on Fraunhofer diffraction using the algorithm proposed in the paper. By this algorithm, the *x* and *y* components of strain have been calculated from the measured strain/stress with an estimated maximum error value of 0.6% and a gauge factor of up to 10.

## Methods

PDMS (10:1) films with thickness of about 1 mm were prepared using a spin coating process in a clean room area. The PDMS sample was square with a side length 2.5 cm. The PDMS was prepared on a PET film, which was used then as structural support and guarantees PDMS flatness during and after processing.

Figure 1 illustrates the fabrication process of a double-side orthogonal grating structure, which involves five steps. As shown in Fig. 1a, wrinkled SiO_x_ layers were formed using O_2_ plasma treatment of a pre-strained PDMS substrate (IoN Wave 10, PVA-TePla, Germany)^25^,^26^. This process was repeated on the other side of the PDMS, with an angle difference of 90°, to form the orthogonal grating structures on both sides of the PDMS, as shown in Fig. 1b[Fig f1]d.

The surface geometry of the grating arrays were characterized using Atomic Force Microscopy (AFM, CSPM5000, Being Nano-Instrument Ltd., China) and a periodicity of about 2 μm on both sides was measured as shown in Fig. 1e[Fig f1]f.

The measurement setup was shown in Fig. 2c. A 532 nm laser (Cobolt 04–01, Cobolt, Sweden) was used as the laser source. The diffracted spots were projected on a black screen. The image was taken by a CCD (E-M1, Olympus Corporation, Japan).

## Additional Information

**How to cite this article**: Guo, H. *et al.* Vectorial strain gauge method using single flexible orthogonal polydimethylsiloxane gratings. *Sci. Rep.* **6**, 23606; doi: 10.1038/srep23606 (2016).
